# Gender trait preferences among smallholder cowpea farmers in northern Ghana: lessons from a case study

**DOI:** 10.3389/fsoc.2023.1260407

**Published:** 2023-10-12

**Authors:** Alhassan Nuhu Jinbaani, Emmanuel Yaw Owusu, Abdul-Razak Mohammed, Theophilus Kwabla Tengey, Michael Mawunya, Francis Kusi, Haruna Mohammed

**Affiliations:** CSIR-Savanna Agricultural Research Institute (SARI), Tamale, Ghana

**Keywords:** cowpea breeding, gender-responsive breeding, trait preferences, gendered institutions, production constraints, gender lens

## Abstract

**Introduction:**

This case study reports on how a gender responsive breeding program contributes to meeting the trait preference of men and women for improved cowpea varieties in northern Ghana.

**Methods:**

Fifty-eight early-maturing, medium-maturing and dual-purpose cowpea lines were planted at the CSIR-SARI research fields and women and men farmers invited for participatory plant breeding (PPB) in 2016. Selected lines from the PPB were further evaluated in 2017 using participatory varietal selection (PVS) in 5 districts in northern Ghana. In addition, 20 focus group discussions (FGDs) were held in 2018 in 10 randomly selected communities with 260 participants (130 women and 130 men) across the districts where the PVS had been held previously.

**Results and discussion:**

The study finds drought tolerance, short cooking time and pest resistance to be the most preferred cowpea traits among both men and women. The study also finds that gender differences exist in trait preference, especially for traits such as seed coat color, earliness, pod above canopy and indeterminate growth habit. As breeding programs focus on improving genetic gains for tolerance of biotic and abiotic stresses, equal attention must be given to breeding for traits desired by women.

## 1. Introduction

### 1.1. Why plant breeders in Ghana paid attention to gender

Before 2015, cowpea [*Vigna unguiculata* (L.) Walp.] breeders in northern Ghana had uneven success introducing improved crop varieties to local smallholders, especially women. Cowpea was considered a woman’s crop, because they did most of the work, from planting to processing. Yet men kept most of the money when cowpeas were sold. After 2015, social scientists joined plant breeders in field research to see what traits farmers valued in cowpeas, and if men and women had different perspectives. As part of this work, the researchers realized that women failed to adopt new cowpea varieties for social reasons, e.g., lack of land, and cash. Women had no access to new seed. In villages where these constraints were addressed, women and men did begin to adopt new cowpea varieties.

Cowpea is one of the most important grain legumes in semi-arid Africa, Latin America and Asia, due to its contributions to food and nutritional security, revenue for smallholder farmers and other value chain actors (Boukar et al., 2019; Dakora and Belane, 2019; Shyam, 2019; Carvalho et al., 2022). Cowpea has a high protein content which ranges from 23% to 32% with high levels of essential amino acids (Muñoz-Amatriaín et al., 2017; Kebede and Bekeko, 2020). In addition, the grain contains iron and zinc (Boukar et al., 2012), crucial for women and children who are deficient in these essential micronutrients on a global level (Olson et al., 2021). In northern Ghana, cowpea is considered a woman’s crop, as it is mostly grown by female smallholders, who depend on it for much of their livelihood (Padmanabhan, 2007). Women provide most of the labor for cowpeas, from planting through weeding, harvesting, transport, threshing, bagging and marketing.

In recent years, various constraints have led to the decline of cowpea productivity in northern Ghana. Farmers achieve only 50% of the potential yield of 2.50 t/ha [Ministry of Food and Agriculture (MoFA), 2016]. Women’s trait preferences are not being met by breeding programs. Recent empirical evidence points to earliness, white seed coat color, large seed size, short cooking time, good taste and high yield as the most preferred traits of cowpea in Ghana and sub-Saharan Africa (Quaye et al., 2009; Salifou et al., 2017; Herniter et al., 2019). However, these studies ignored preferences by gender. Agricultural technologies are not gender neutral (Polar et al., 2017). Men and women may have different reasons for adopting a new agricultural technology.

Male farmers usually achieve higher yields of cowpea than females, who have less access to land, credit and other resources. Because of these constraints, women are also less likely to adopt improved cowpea varieties. Releasing crop varieties that meet gender trait preferences may not necessarily lead to adoption, if these constraints persist. Adoption requires community and institutional changes, including new farming behavior and rethinking rural attitudes and norms. These concerns have driven breeding programs to adopt gender research on the trait preferences of men and women. However, breeding programs must also leverage community institutions to achieve widespread adoption of improved cowpea varieties.

### 1.2. Context

The Council for Scientific and Industrial Research—Savanna Agricultural Research Institute (CSIR-SARI) has the mandate to develop technologies for increased agricultural productivity for improved food security, nutrition and livelihoods of smallholder farm households in northern Ghana. However, in the past, improved seed from breeding programs and other technologies were generated without integrating gender, even though men and women farmers have different needs, and unequal access to technology, resources and opportunities (Quisumbing et al., 2014).

A muti-disciplinary team of two breeders and two socio-economists (with specializations in gender studies, monitoring, and evaluation and agribusiness management) implemented the Tropical Legume III (TLIII) Project, which targeted men and women farmers, processors, consumers and traders who were mostly small-scale. Before 2015, the cowpea improvement program consisted mainly of breeders. However, there was the realization that breeding was targeted towards end-users with different needs and that technology transfer was complex, and that specialized skills were called for. This was when the social scientists were included into the cowpea improvement program in 2015, and the program began to engage more with farmers. The project also collaborated with the Seed Producers Association of Ghana (SEEDPAG) and Heritage Seeds, a private seed producing company, to produce certified cowpea seed for farmers. SEEDDPAG and Heritage Seeds also mentored and provided technical assistance to multi-stakeholder platforms (MSPs) under the community seed production.

Recent literature reports breeding programs incorporating trait preferences of both men and women, e.g., for improved cassava in Nigeria, and for beans in Kenya and Uganda (Tufan et al., 2018). However, the role of gendered institutions in breeding, dissemination and adoption is seldom reported in the literature. The gendered institutions are MSPs, village savings and loan associations (VSLAs), community seed production and farmer participatory varietal selection (FPVS). They are called “gendered institutions” because of the deliberate efforts made by the breeding program to include women, who were previously excluded. The case study documents the experiences of implementing a gender-responsive breeding program over five years from 2015 to 2019. This study included gendered preferences for cowpea traits, access to land and the role of informal institutions as part of the social targeting and demand analysis stage of the breeding process, as explained by Tufan et al. (2018). The social targeting and demand stage is incomplete without considering the gender analysis framework, consisting of six interdependent, interacting components: assets, markets, information, risks, institutions and policies. The effect of one component, for example, assets, needs to consider the influence of the others (Tufan et al., 2018). These components may in turn affect breeding stages. For example, a cowpea improvement program may target women’s trait preferences, only to find that varietal adoption is still be stymied by local institutions that restrict women’s access to land.

## 2. Analysis

### 2.1. Research and information generated on gender

In 2015, the Cowpea Improvement Program of CSIR-SARI was redesigned to respond to the gendered needs of smallholder farmers through the Tropical Legume III (TLIII) Project, with support from the International Crops Research Institute for the Semi-Arid Tropics (ICRISAT) and the International Institute of Tropical Agriculture (IITA). Improved cowpea varieties were introduced, mainly to women smallholders, to improve cowpea productivity and reduce the gender yield gap. Women were organized in groups or encouraged to join multi-stakeholder platforms (MSPs), to gain access to the new seed. The MSPs produced community seed to sell to their members and other farmers. Some of the women’s groups also operated VSLAs. The MSPs and the VSLAs, herein referred as gendered institutions, were created by the breeding program to help overcome gender-based constraints, for example, women’s poor access to land, cash and labor. Field demonstrations and farmer field days were used to disseminate cowpea varieties with farmers (including women’s groups) in the Guinea and Sudan Savanna agroecological zone of northern Ghana, which is prone to drought and poverty (Antwi et al., 2015). CSIR-SARI collaborated with the Ministry of Food and Agriculture (MoFA), which provided extension services to introduce the improved cowpea varieties to farmers.

Social scientists were brought on board the crop breeding program in 2015. In 2017 farmer participatory varietal selection (PVS) revealed which traits men and women preferred in cowpea varieties. Focus Group Discussions (FGDs) were organized in December 2018 to validate these cowpea trait preferences. From the FGDs, it was found that women were not adopting new varieties because of various gender-based production constraints.

There was a training by the GREAT program for biological and social scientists in 2018 on how breeding programs could be gender-responsive for agricultural transformation (Mangheni and Tufan, 2022). The Bill and Melinda Gates Foundation (BMGF) requested this training for scientists implementing BMGF-funded programs, to better incorporate gendered needs into breeding objectives.

By incorporating gender and hiring social scientists, the breeding program became more engaged with farmers, starting institutions within the communities (MSPs, VSLAs) and engaging with local authorities to improve breeding efficiency and to promote new cowpea varieties for men and women. Social scientists helped breeders incorporate gender with: yield trials, on-farm trials, demonstration fields and the promotion of the newly released varieties. The breeding team prioritized breeding objectives that addressed women’s needs. For example, women prefer traits that make a good soup or a cowpea variety that produces pods above the canopy for easier harvesting.

The CSIR-SARI cowpea improvement program needed to help communities overcome agronomic constraints such as poor soil fertility, pests, and drought (Salifou et al., 2017) and institutional issues such as access to credit, and the high cost of labor and other inputs (Akudugu et al., 2012; Bashir et al., 2018). New institutions were established, for example MSPs in Manga in the Upper East Region, and Tumu in the Upper West Region, to give women and men access to seed and credit. The cowpea improvement program also considered social constraints that limit the adoption of cowpea varieties. The program involved local authorities and opinion leaders to improve women’s access to land and to give both genders equal opportunities and benefits.

### 2.2. How attention to gender influenced the breeding initiative

To improve the adoption of new legume varieties among women and men farmers, the Tropical Legume III project integrated gender responsiveness into the breeding program from 2015 to 2019 through ICRISAT and IITA, implemented by national agricultural research systems (NARS), including CSIR-SARI. Before 2015, the cowpea improvement program was gender-neutral. In the early 2000s, yield, resistance to drought, insect pests and striga (a parasitic weed) were major concerns for breeders in Ghana and in most national breeding programs in sub-Saharan Africa (Singh et al., 2002). Many breeding programs did not include social scientists, and failed to take into account the trait preferences of the men and women who would use the new cowpea varieties.

From 2007, with help from IITA, breeding efforts in Ghana were geared towards producing cowpea varieties tolerant for drought, heat, aphids and striga (Boukar et al., 2019). By 2015 and onwards donor agencies and the Consortium of International Agricultural Research Centers (CGIAR) encouraged national breeding programs to be demand-driven and to focus on the preferences of the market and of farmers.

Before 2015, breeding approaches such as field trials, mother-and-baby trials, farmer field schools and demonstrations were established practice at the cowpea improvement program at CSIR-SARI. However, these gender-blind approaches rarely considered the needs of men and women. This changed when social scientists joined the breeding program in 2015. The program began using gender-responsive methods, such as participatory plant breeding (PPB), and participatory varietal selection (PVS): experimenting with farmers in organized groups to improve breeding efficiency. Women farmers participated, but they seldom invited to become lead farmers, hosting field demonstrations.

From 2016 to 2018, field trials, farmer participatory varietal selection (PVS) and focus group discussions were designed to consider gender and to increase the adoption of new cowpea varieties. The breeding program began to identify constraints faced by men and women. There was also a deliberate effort to implement breeding activities that would benefit women. With advice from the social scientists, the program began to use FGDs, allowing researchers to identify men and women’s perspectives of constraints and cowpea trait preferences. The FGDs revealed that women had almost no access to land, or credit, and nowhere to get improved seed. Women were also less represented in the multi-stakeholder platforms (MSPs), which the project had previously set up.

To help women cowpea farmers overcome institutional constraints, which were reinforced by local norms and values, the breeding team established gendered institutions such as VSLAs for women, while renewing the emphasis on MSPs. The program maintained its primary role of breeding to overcome biotic and abiotic production constraints, but also began to consider that women’s access to land could be improved by engaging with village chiefs and opinion leaders, who have a role to play in disseminating cowpea varieties. After the FGDs showed that few women took part in the multi-stakeholder platforms (MSPs), the program increased female membership in the MSPs to 30%–50%. The MSPs went into community seed production with the help of the breeding team, to improve access to improved seed. Five MSPs on average produced 1 metric ton of quality declared seed (QDS) which was sold to members at reduced prices and to farmers outside the MSPs. The funds from the sales went directly to the MSPs. Heritage Seeds, a locally registered seed company and SEEDPAG, who were partners in the TLIII Project, provided technical assistance to the MSPs.

The cowpea breeding team established village savings and loan associations (VSLAs), mostly as women’s groups, to make small loans. The breeding program popularized already released varieties (Wankae, Kirkhouse Benga, and Padi-Tuya) through demonstrations, experiments conducted by organized groups and community seed production. These varieties were chosen because they actually did meet men and women’s trait preferences, as documented during the PVS in 2017 and FGDs in 2018. All of these varieties were white, except Kirkhouse Benga which was white with purple marks. They were resistant to striga and moderately drought-tolerant. They were early maturing (on average, 65 days) and on most of them, their pods grew above the canopy. The breeding team made efforts to involve women in all the project activities; sometimes the men were even outnumbered. This increased adoption of improved cowpea varieties among both men and women in northern Ghana (Wahaga, 2019; Adams et al., 2021). The methods used to pay specific attention to gender are shown in Table 1. In the project communities 50% to 60% of farmers adopted the promoted varieties: Wankae, Kirkhouse Benga, and Padi-Tuya.

**Table 1:** Methods and approaches used in the case study.

Activity	Paid specific attention to gender?
Participatory plant breeding (PPB)	Yes
Participatory varietal selection (PVS)	Yes
Mother-baby trials	Yes
Farmer-experimenters formally organized in groups, committees or networks to contribute to breeding	Yes
Study of trait preferences	Yes
Farmer-to-farmer visits or exchanges	Yes
Farmer field school experiments or demonstrations	Yes
Other activities, VSLAs and MSPs	Yes
Focus group discussions (FGDs)	Yes

The study findings that women preferred brown seed coat, indeterminate growth and pods that grow above canopy were incorporated into the ongoing Accelerated Varietal Improvement and Seed Delivery of Legumes and Cereals in Africa (AVISA) project, which started in 2019 and has led to the release of two cowpea varieties (SARI-tuya and Tuzievellenga). SARI-tuya has large, white seeds, is high yielding, has pods above the canopy and is resistant to striga, all traits shown by the gender research to be preferred by men and women. Tuzievallenga was developed in response to women’s preference for a brown, early maturing variety; it is also high yielding and resistant to striga, traits that men also value. Both of these new varieties have high iron and zinc content, a trait not mentioned by farmers, but included by the breeding team to meet the nutritional requirements of women and children in northern Ghana. In some instances, the stated preference for good taste reflects a craving for iron and zinc. Some of the best performing genotypes now in breeding pipeline include the traits preferred by women.

### 2.3. Methods and approaches: advantages and shortcomings

The cowpea improvement program began using participatory plant breeding (PPB) in 2016, farmer participatory varietal selection (PVS) in 2017 and FGDs in the monitoring survey in 2018.

#### 2.3.1. Participatory plant breeding

Fifty eight (58) early-maturing, medium-maturing and dual-purpose cowpea lines received from IITA were given a preliminary evaluation at the CSIR-SARI research field. The lines were planted and women and men farmers were invited for participatory plant breeding (PPB) in 2016. PPB refers to the entire process of setting breeding objectives, making crosses, developing and releasing improved varieties and suppling basic seed classes to growers (Ashby, 2009). With the PPB, the breeders made the selection on what lines would be advanced to next breeding stage based on both the suggestions and preferred traits from farmers. Thus, the context of PPB in this study emphasizes selections made due to suggestions from farmers. The PPB was limited to only farmers and they were 20 in number: 10 men and 10 women. Four of the women were also processors. Two of the men were also traders, in addition to being farmers. The PPB process finally led to the selection of 20 promising lines based on the participants’ preferred traits. On average, 12 field visits were made by scientists together with the farmers during PPB activities in the 2016 cropping season. These number of field visits made the PPB costly and time consuming, confirming the conclusion of Witcombe et al. (1996) that PPB is costly and time consuming. The other limitation of PPB was that the stakeholders were not involved in the development of these lines since the lines were brought from IITA, as noted earlier.

#### 2.3.2. Participatory varietal selection

The selected lines from the PPB were further evaluated in 2017 with the key cowpea stakeholders (men and women farmers, women processors, men and women traders, agricultural extension staff and agro-input dealers) in advanced yield trial (on-station) via participatory variety selection (PVS). In the context of this case study, PVS exclusively refers to farmers taking part in the evaluation of finished improved varieties based on their preferences from a set of suitable choices before release to the general public, as explained by Witcombe et al. (1996) and Ashby (2009). Fourteen promising lines were selected for multi-location trials, using the mother-and-baby approach at five locations: Nyankpala, Yendi, Manga, Damongo, and Tumu. The locations were purposively selected to represent the agro-ecologies in northern Ghana in order to have research results that is stable and consistent across different agro-ecologies. Nyankpala and Yendi in the Northern and Tumu in the Upper West regions represent the guinea savanna. Manga in the Upper east region represents the Sudan-savanna. Damongo in the Savanna region represents the transitional zone. A randomized complete block design with three replications was used at all five locations for two years (2016 and 2017). One hundred and fifty (150) women and 90 men cowpea value chain actors in 2017 attended the PVS (Table 2). The PVS participants selected varieties based on grain yield, maturity, pod length, grain color, grain size, and biomass yield.

**Table 2:** Traits selected by cowpea producers in PVS across the five study location.

	Percent of farmers (men and women) selecting each trait by study location															
Trait	Tumu		Manga		Damongo		Nyankpala		Yendi		Total					
Gender	Female	Male	Female	Male	Female	Male	Female	Male	Female	Male	Female	%	Male	%	Grand total	%
White seed coat	29	11	21	13	25	30	25	14	12	15	112	74.7	83.0	92.2	195.0	81.3
Brown seed coat	13	1	7	2	5	2	6	0	7	2	38	25.3	7.0	7.8	45.0	18.8
Early maturing	35	8	23	11	26	25	27	10	16	13	127	84.7	67.0	74.4	194.0	80.8
Medium maturing	7	4	5	4	4	7	4	4	3	4	23	15.3	23.0	25.6	46.0	19.2
Large seed size	33	11	23	15	24	30	24	13	15	16	119	79.3	85.0	94.4	204.0	85.0
Medium seed size	9	1	5	0	6	2	7	1	4	1	31	20.7	5.0	5.6	36.0	15.0
Taste	42	12	28	15	30	32	31	14	19	17	150	100.0	90.0	100.0	240.0	100.0
Medium cooking time	0	0	0	0	0	0	0	0	0	0	0	0.0	0.0	0.0	0.0	0.0
Short cooking time	42	12	28	15	30	32	31	14	19	17	150	100.0	90.0	100.0	240.0	100.0
High grain yield	37	9	16	9	27	25	30	13	15	14	130	86.66	70.0	77.8	193.0	83.33
High biomass yield	5	3	12	6	3	4	3	1	0	3	20	13.0	20.0	22.2	40.0	16.66
Indeterminate	38	8	21	11	25	15	23	4	13	14	120	80.0	52.0	57.8	172.0	71.7
Determinate	4	4	7	4	5	17	8	10	6	3	30	20.0	38.0	42.2	68.0	28.3
Pod above canopy	40	9	16	10	26	25	30	9	14	13	126	84.0	66.0	73.3	192.0	80.0
Pod within canopy	2	3	12	5	4	7	1	5	5	4	24	16.0	24.0	26.7	48.0	20.0
Insect pest resistance	42	12	28	15	30	32	31	14	19	17	150	100.0	90.0	100.0	240.0	100.0
Drought tolerance	42	12	28	15	30	32	31	14	19	17	150	100.0	90.0	100.0	240.0	100.0

Farmer Participatory Varietal Selection (FPVS) Survey, 2017. NB. number of women = 150; number of men = 90; population of participants (grand total) = 240.

PBB and PVS are fairly similar approaches, which efficiently document farmers’ preferences for certain traits and varieties. PVS can be easily set up to gauge the different responses of men and women. However, these methods reveal little about the institutional constraints that hinder adoption of new varieties. Subsequently focus group discussions were held in the study areas.

#### 2.3.3. Focus group discussions

The breeders and social scientists held 20 focus group discussions (FGDs) in 10 communities that were randomly selected with 260 participants (130 women and 130 men) across the districts where the PVS had been held in 2017. The participants to the FGDs were also randomly selected as random sampling provides better estimate of parameters in a study compared to purposive sampling (Singh and Masuku, 2014). These districts were Yendi and Tolon districts in the Northern region, Bawku East in the Upper East region, Damongo in the Savanna region, and Sissala East in the Upper West region, as shown in Table 3. After each FGD, data were taken on each participants’ adoption of any improved cowpea variety, total output, land ownership, access to land, education, land area under cowpea production and age, as described in Table 4.

**Table 3:** Cowpea traits ranked by men and women in FGDs (in order of preference).

Region	District	Men	Women
Northern region	Yendi	Pest resistance High yielding Good taste Drought tolerant Shorter cooking time Larger grain size Early maturing White seed coat Higher fodder	Drought tolerant Higher yielding Pest resistance Early maturing Shorter cooking time Good taste Larger grain size Indeterminate growth habit Brown seed coat White seed coat Pod above canopy
	Tolon	Pest resistance High yielding Good taste Drought tolerant Shorter cooking time Larger grain size Striga tolerance	Drought tolerant Higher yielding Pest resistance Early maturing Striga tolerance Shorter cooking time Good taste Larger grain size Indeterminate growth habit
Upper east	Bawku east	High yielding Pest resistance Good taste Drought tolerant Striga tolerance	Shorter cooking time Good taste Brown seed coat Higher yielding Striga tolerance Pod above canopy
Savanna region	Damongo	Short cooking time High yielding Pest resistance Good taste Striga tolerant Drought tolerant Early maturing	Shorter cooking time Good taste Higher yielding Pest resistance Larger grain size Indeterminate growth habit Brown seed coat
Upper west	Sissala east	High yielding Pest resistance Good taste (3rd) Striga tolerant Larger grain size Drought tolerance Pod above canopy	Pest resistance Shorter cooking time Good taste Brown seed coat Higher yielding Striga tolerance Pod above canopy

Focus Group Discussions (FGD), 2018.

**Table 4:** T-statistic mean differences between subsidy and non-subsidy households.

Variable	Description	Male respondents: mean	Female respondents: mean	Difference
Adoption status	1 if a respondent adopted any improved cowpea variety; 0 otherwise	0.275	0.108	0.167^***^
Total output (Kg)	Total yield a farmer obtained in Kg	2750	1900	850^***^
Land_ownership	1 if respondent owned land; 0 otherwise	0.908	0.225	0.683^***^
Access_land	1 if respondent had access to land; 0 otherwise	0.942	0.392	0.55^***^
Education	1 if respondent has at least basic education; 0 otherwise	0.475	0.192	0.283^***^
Land area (Acres)	Land area under cultivation	2.00	1.267	0.733^***^
Age	Age of respondent in years	40.31	38.93	1.38

Focus Group Discussions’ (FGDs) participants individual-level data, 2018. NB. ^***^, ^**^, and ^*^standard for 1%, 5% and 10% at statistical significance. These are the power of significance or confidence levels upon which the data must be interpreted.

The interactive nature of FGDs enabled participants to validate responses from one another (Powell and Single, 1996) and let the breeding team investigate participants’ behaviors and motivations (Carey and Smith, 1994). FGDs not only reconfirmed the results from the PVS, they also led to the discovery of agronomic and institutional constraints faced by men and women in cowpea production. Qualitative data were collected from respondents through the FGDs held between 12th to 28th December 2018. There were separate FGDs for men and women, to allow the women to express themselves freely. Smaller groups were chosen because larger FGDs are challenging to manage and they limit each participant’s opportunities to speak (Morgan, 1997). The interviewers consisted of a lead facilitator, two note-takers and an observer. The lead facilitator was a social scientist. The note-takers were another social scientist and an agricultural extension officer. The observer was a breeder. The lead facilitator would ask for permission to take pictures and make audio recordings. Participants invariably consented.

During the interviews the lead facilitator asked the questions. The note takers wrote down the responses. The observer took notes on the clarity of the questions asked, the validity of the responses, body language and eye contact of the lead facilitator and the participants and whether the participants were comfortable with the venue, the questions asked and the general atmosphere of the meeting. After each FGD, the facilitators would then meet to go through the responses, compare notes and validate the responses. The data was then entered into an excel file for analysis. The qualitative data was organized around themes, and sometimes included direct quotes from the interviews. Reporting qualitative data this way makes the findings comprehensive and clearly presents their typicality (Rubin and Rubin, 1995). The recordings were transcribed, which made it possible to recover the respondents’ exact phrases during data entry and to analyze their responses qualitatively and descriptively (Bertrand et al., 1992).

## 3. Changes in the breeding process and practice because of learning about gender

For years, the team consisted only of breeders and field technicians. In 2015, social scientists with expertise in gender and agribusiness joined the program, which began to breed varieties for men and women who may have different preferences for cowpea traits. Crucially, the breeders joined social scientists in the field studies in 2017 and 2018. This not only helped to build team spirit, but also ensured that the breeders accepted the study conclusions.

In addition to organizing PVS, desired traits were also differentiated by gender, and by region. For example, women in the Northern region considered drought tolerance as most important whereas women in the Upper East and Savanna regions wanted short cooking times (Table 3). This finding has influenced the breeding program to always conduct PVS and FGDs on trait preferences across regions since 2019. Traits are now being selected to target particular regions. The variety design always includes both agronomic and consumer traits for wide user acceptability. The choice of materials to advance to the next stage of breeding is then based on the different preferences of men and women. Thus, lessons from PVS and FGDs now feed into organizing PPB. Women are now among the lead farmers when new materials are evaluated on-station or on-farm.

The definition of markets and the strategies for seed multiplication and dissemination also changed. MSPs were trained to produce quality declared seed which they sold to their members and to neighbors. Women farmers became more involved in community seed production. Farmer groups and VSLAs were formed in the study communities to empower women, who gained access to credit. Two cowpea varieties were finally released in November 2022 that had been developed in response to gender related-concerns.

## 4. Breeding outcomes and impacts

### 4.1. PVS and preferences for traits of improved cowpea varieties

During the PVS, men and women farmers in the five locations expressed their preferences for specific cowpea traits (Table 2). All of the 150 females and the 90 males preferred drought tolerance, short cooking times and pest resistance (Table 2). Most farmers selected grain yield (83.33%), but was preferred by slightly more women (87%) than men (79%) (Table 2).

Several other traits were selected by most farmers, with men and women mainly in agreement. For example, 85% of all farmers selected large seed size: 79% of the women and 94% of the men. White seed coat, selected by most farmers (81%), was more chosen by more men (92%) than women (75%). Early maturity was selected by most farmers (81%), but was important to a few more women (85%) than men (74%). Pod above canopy was also important to most farmer (80%), especially to women (84% vs. 73% for men). Women and men generally prefer the same traits, with some slight differences, especially for characteristics with market importance.

More women preferred pods that grow above the canopy, because they are easier to harvest, a task generally done by women. Women also preferred indeterminate growth habit because such varieties provide fresh leaves to make soup and other dishes. Some women respondents (25%) preferred brown seed coat to prepare *waakye* (rice and cowpea cooked together) and red-red (boiled cowpea with palm oil eaten with fried plantain). The women say that brown cowpeas are cheaper than white ones and can be used for home-made flour which is prepared with sprouted cowpea seeds; the seed coat is removed during processing, so its color does not matter. All the cowpea varieties released by CSIR-SARI before implementing gender responsive breeding were white, but based on the results from this preference studies, one of the two cowpea varieties approved by National Variety Release and Registration Committee for release in November, 2022, Tuzievallenga, is brown and there are several others in the pipeline.

Early maturity was the fifth most selected trait of the 16 evaluated by farmers (Table 2). Female respondents were slightly more likely than males to prefer early maturing varieties. Some men preferred medium-maturing varieties, which were perceived to yield more. Women farmers said that early maturing varieties would allow multiple cropping per year, while also escaping terminal drought, which in today’s changing climate is now more common in northern Ghana.

### 4.2. Focus group discussions

The sex-disaggregated focus group discussions (Table 3) supported the team’s conclusions about trait preferences from PVS (see the previous sub-section). Across the regions, men and women gave attention to agronomic traits (insect pest resistance, high yield and drought tolerance), and consumer traits (shorter cooking time and good taste). These traits are must-haves for any new improved cowpea variety.

Specific to certain districts, gender differences in cowpea trait preference for women were shorter cooking time in Bawku East in the Upper East region and larger grain size in Damongo in the Savanna region. In Upper East men preferrene was pest resistance while women valued shorter cooking time (Table 3). Spraying to control pests is done mainly by men, but they would like to spray less often, which they now do on average five times a season. Faster cooking cowpeas would give women more time for other activities, while using less fuel wood (Martey et al., 2022).

Results from the individual-level data of the FGDs’ participants revealed statistically significant differences between men and women cowpea farmers in terms of adoption, total output, land ownership, access to land and land area under cowpea cultivation in Ha, as indicated from the two-sample t-statistic mean differences in Table 4. There was, however, no systematic difference between men and women in terms of age. According to Moore and Kirkland (2007), *p-values* in the *t table* for the two-sample t-statistic method are accurate when the sizes of the two samples being compared are equal. In addition, the two-sample t-statistic mean differences are more robust than one sample t-statistic methods, and robust also against non-normality. From Table 4, the level of adoption among men cowpea farmers was about 28% as against about 11% among women cowpea farmers. Total output, on average, among men cowpea farmers was higher (2750Kg) compared to women cowpea farmers (1900Kg). Conversely, land under cultivation, on average, was also greater among men (2Ha) compared to women (1.267Ha). It suffices to say that the low adoption of cowpea improved varieties and low output among women as compared to men in the sample could be due to land ownership, access to land and education that favour men relative to women, as shown in Table 4. Though the two-sample t-statistic mean differences do not imply causal effects, FGDs of both men and women confirmed this finding.

## 5. Gender constraints in cowpea production

Women were not rejecting new cowpea varieties for their genetic traits; women were facing extreme social barriers to adoption, as women participants explained in the sex-disaggregated focus group discussions. For example, from the discussions, it came to light that land ownership across the studied communities was by inheritance. Male farmers inherit farmland from their fathers. However, all community lands are entrusted to the chief of the community. Few farmers borrow land. One man said:

“One can also borrow or beg land to farm, without necessarily owning land. You can in turn give a portion of your produce to the landowner after harvest. Women in the community have access to land, but this is based on the decision of their husbands. Women in this community mostly grow okra and pepper. That is why we do not allocate lands permanently to them. These crops also enrich the soil. After a time, we collect back the lands to grow cereals and allocate different lands to the women. We men have the responsibility to feed the family, not the women.”

This suggests that women not only have little access to land, but they are sometimes loaned a field so that they can enrich the soil for a following cereal crop grown by men.

Another man said:

“Over here there is not enough land to cultivate maize, so we do not allocate lands permanently to women. Therefore, when the man is in need of land, there are fewer challenges taking the lands back from the women.”

Women only have access to land through their husbands. This is a major constraint to the adoption of improved cowpea varieties. Poor access to land explains why it has always been difficult to get women as lead farmers to host demonstrations, which the breeding team did not realize until this study. Some of the women said: “Our husbands control all the land.” Societal norms tend to privilege men, given them more power over women, and allowing men to wield more control over assets and resources (Sen, 1990; Pérez et al., 2015). The FGD revealed that few men, and no women, had access to improved seed. Few claimed to get improved seed from CSIR-SARI through field demonstrations. They explained that unlike maize, they do not buy improved cowpea seeds at agro-input shops, and that cowpea seed is expensive.

Men and women either use recycled seed from the previous harvest or they plant grain they buy in the market. Males and females have equally poor access to improved seed. Astonishingly, the FGDs revealed a lack of representation of women in the MSPs that were established by the cowpea breeding team. After learning this, the program took steps to involve women in the platforms, which also began to produce seed that was available in the study communities (see the previous sub-section: How attention to gender influenced the breeding initiative).

Men take more of the cash from the sale of cowpea, claiming that this is because they take care of the whole family. Most women do not sell their cowpea. They produce it to feed the family. They only sell during difficult times when they need money. Even when women have to sell, they are obliged to inform their husbands. Women and children provide labor for all the field activities, including planting, harvesting and fetching drinking water for the workers. Household gender relations seriously affect the intra-household distribution of income (Farnworth, 2011). Men and women may collaborate to bring wealth into the family, but the men often take most of the money (Sen, 1990). In response to this finding, the team organized VSLAs to give women access to credit (discussed above).

## 6. Discussion

The coming together of breeders and soil scientists in a breeding program offers them the opportunity to develop market-driven breeding objectives that meet the needs of end-users, including men and women. According to Tiwari et al. (2022), this includes matching breeding goals to product performance for each segment of end-users or market. The finding from the study that drought tolerance, short cooking times and pest resistance (Table 2) were preferred by both men and women is consistent with that of Karikari et al. (2023). Karikari et al. (2023) found that farmers in the Upper West region of Ghana preferred cowpea traits that are drought tolerant, pest resistant and short cooking time. The implication is that cowpea farmers are adapting to climate change and variability through the choices they make regarding trait preference. These choices include the preference for early maturing cowpea trait. From the study, the finding that female respondents were slightly more likely than males to prefer early maturing varieties indicates that women farmers as well, are increasingly becoming conscious of the negative impact of climate change and variability and are willing to adapt. Rabé et al. (2022) also found early-maturity as a preferred trait among cowpea farmers in Niger. Unlike this study, Rabé et al. (2022) did not look at trait preferences between men and women cowpea farmers. These findings are supported by the conclusion of Moussa et al. (2023) that farmers’ strategies for tackling climate change and variability in Southern Niger included adopting early maturing varieties with high yield and tolerant to drought, pests, and diseases. Increased urbanization, coupled with the growing market for cowpea in Ghana has also influenced the preference for a consumer trait such as less cooking-time among cowpea producers and consumers (Mishili et al., 2009).

As observed in Table 2, most farmers selected grain yield (83.33%), but was preferred by slightly more women (87%) than men (79%). Similar studies have consistently found preference for high grain yield among cowpea farmers (e.g., Rabé et al., 2022; Karikari et al., 2023; Moussa et al., 2023). These studies (Rabé et al., 2022; Karikari et al., 2023; Moussa et al., 2023), however, assumed preference for traits among producers and end-users to be gender-blind, therefore did not consider trait preferences for both men and women. From the study, the finding that more women preferred pods that grow above the canopy for the reason that such cowpea are easier to harvest confirms (Ridgeway, 2009; Sylla et al., 2023) that gender relations fit into social structures that define agricultural tasks. Such tasks such as harvesting of cowpea as generally done by women may also define trait choices by women farmers. Women also preferred indeterminate growth habit because such varieties provide fresh leaves to make soup and other dishes. This finding confirms that of Horn et al. (2015) and Owade et al. (2020) who found farmers using fresh cowpea leaves and pods as vegetables. These vegetables are rich in minerals including vitamins and iron which are deficient in sub-Sahara Africa. This makes cowpea a good crop in fighting against food and nutrition insecurity in SSA (Owade et al., 2020).

From the FGDs, the finding that across regions, producers prefer different traits highlights the importance of considering geographical location in trait development, besides gender. PVS results may be limited by inadequate means of verification. Sex disaggregated focus group discussions can provide such means of verification and validating results from PVS. A similar approach was employed by Kyebalyenda et al. (2022) in studying preferred genotypes and different sensory attributes in cowpea for vegetable use in eastern Uganda. Across the regions, the FGDs highlight how men and women gave attention to agronomic traits (insect pest resistance, high yield, and drought tolerance), and consumer traits (shorter cooking time and good taste).

The observation from the sex-disaggregated focus group discussions that women had limited access to land can be considered as one of the social barriers to adoption in northern Ghana. The nexus between gender relations and social structures also define access to land and other agricultural inputs (Ridgeway, 2009; Sylla et al., 2023). Rules embedded in communities regulate activities, decision and roles mostly in favour of men to the disadvantage of women (Kabeer, 1994). The involvement of traditional and opinion leaders in breeding programs in local communities is therefore critical in promoting access to and ownership of land by women.

### 6.1. Good practices

Participatory varietal selection allows farmers and other actors on the cowpea value chain to select their preferred lines (Horn et al., 2015). Because the breeding team implemented PVS with gender in mind, the improved varieties were accepted by women and men end-users. Because the PVS were participatory, lines that were rejected became good materials for crossing with desirable lines during the PPB stage, which gets the genotypes right from the onset so that they meet the expectations of men and women end-users. As the PVS became more gender-sensitive, they gave a sense of ownership to participating women’s groups and a sense of accountability to the breeding team. The limitation of PVS is that it does not offer the participants an opportunity to explain themselves and to query one another, unlike focus group discussions (FGD).

Separate FGDs for men and women in 2018 allowed them to discuss their trait preferences. The breeding team became more enlightened on both agronomic and consumer trait preferences. Subsequent varietal designs and evaluations in on-farm trials and field demonstrations since 2019 have focused on the information obtained from the PVS and FGDs. This gave both sexes the opportunity to interact well with the breeding team. Involving village chiefs and opinion leaders eased some of the constraints women had previously faced in the project communities. The MSPs with 30%–50% membership of women provided the platform for community seed production. The VSLAs also helped women buy improved seed, mineral fertilizer and to hire labor. There was increased adoption of improved cowpea seeds in farm communities, and there are plans to upscale these novel practices to other districts in northern Ghana to generate wealth and empower women.

### 6.2. Lessons

Women’s unequal access to land, credit and membership in farmer-based organizations were discussed in the communities that took part in the study. These discussions with the chiefs, opinion leaders, local government officials and the research team led to the formation of gendered-institutions (MSPs and VSLAs). Women’s access to farmland, for cowpea and other crops, increased in the intervention communities. The MSPs produced seed of preferred cowpea varieties, specifically, Padi-Tuya and Wang-Kae which have short cooking time. This improved access to improved cowpea seed and adoption of varieties, especially for women farmers. The community seed production, improved access to land by women, the MSPs and VSLAs will remain after TLIII project. Further research on these enduring effects on income, food security and nutrition between male and female-headed households will be worth undertaking in a larger sample size to provide evidence for policy formulation on mainstreaming gender into national breeding programs.

As breeding programs focus on improving genetic gains for tolerance of biotic and abiotic stresses, equal attention must be given to breeding for traits desired by consumers. Gender differences exist, especially for traits such as seed coat color, earliness, pod above canopy and indeterminate growth habit. Breeding varieties with desired traits is not always enough to ensure their adoption. Programs must realize that women cannot adopt new varieties without access to seed, land and money. Gendered institutions such as MSPs, VSLAs, and seed producers’ groups can help to foster the adoption of improved cowpea varieties.

## Data availability statement

The raw data supporting the conclusions of this article will be made available by the authors, without undue reservation.

## Author contributions

AJ: Conceptualization, Data curation, Formal analysis, Funding acquisition, Investigation, Methodology, Project administration, Resources, Software, Writing – original draft, Writing – review & editing. EO: Data curation, Formal analysis, Methodology, Visualization, Writing – review & editing. A-RM: Data curation, Formal analysis, Investigation, Methodology, Validation, Writing – review & editing. TT: Investigation, Methodology, Supervision, Validation, Writing – review & editing. MM: Formal analysis, Investigation, Methodology, Validation, Visualization, Writing – review & editing. FK: Methodology, Supervision, Validation, Visualization, Writing – review & editing. HM: Validation, Visualization, Investigation, Methodology, Writing – review & editing, Supervision.
